# Development of a patient-centred care pathway across healthcare providers: a qualitative study

**DOI:** 10.1186/1472-6963-13-121

**Published:** 2013-04-01

**Authors:** Tove Røsstad, Helge Garåsen, Aslak Steinsbekk, Olav Sletvold, Anders Grimsmo

**Affiliations:** 1 Department of Public Health and General Practice, Norwegian University of Science and Technology, Trondheim, Norway; 2 Department of Health and Welfare Services, City of Trondheim, Trondheim, Norway; 3 Department of Geriatrics, St. Olavs Hospital, Trondheim University Hospital, Trondheim, Norway; 4 Department of Neuroscience, Norwegian University of Science and Technology, Trondheim, Norway; 5 Norwegian Health Net, Trondheim, Norway

**Keywords:** Care coordination, Continuity of patient care, Healthcare disparities, Multi-morbidity, Patient discharge, Primary care, Home care services, Interdisciplinary communication, Organizational culture, Health services for the aged

## Abstract

**Background:**

Different models for care pathways involving both specialist and primary care have been developed to ensure adequate follow-up after discharge. These care pathways have mainly been developed and run by specialist care and have been disease-based. In this study, primary care providers took the initiative to develop a model for integrated care pathways across care levels for older patients in need of home care services after discharge. Initially, the objective was to develop pathways for patients diagnosed with heart failure, COPD and stroke. The aim of this paper is to investigate the process and the experiences of the participants in this developmental work. The participants were drawn from three hospitals, six municipalities and patient organizations in Central Norway.

**Methods:**

This qualitative study used focus group interviews, written material and observations. Representatives from the hospitals, municipalities and patient organizations taking part in the development process were chosen as informants.

**Results:**

The development process was very challenging because of the differing perspectives on care and different organizational structures in specialist care and primary care. In this study, the disease perspective, being dominant in specialist care, was not found to be suitable for use in primary health care because of the need to cover a broader perspective including the patient’s functioning, social situation and his or her preferences. Furthermore, managing several different disease-based care pathways was found to be unsuitable in home care services, as well as unsuitable for a population characterized by a substantial degree of comorbidity. The outcome of the development process was a consensus that outlined a single, common patient-centred care pathway for transition from hospital to follow-up in primary care. The pathway was suitable for most common diseases and included functional and social aspects as well as disease follow-up, thus merging the differing perspectives. The disease-based care pathways were kept for use within the hospitals.

**Conclusions:**

Disease-based care pathways for older patients were found to be neither feasible nor sustainable in primary care. A common patient-centred care pathway that could meet the needs of multi- morbid patients was recommended.

## Background

In Norway, as in most Western societies, health authorities consider health and social services to be fragmented; especially challenging is a lack of continuity of care for elderly and chronically ill patients [1-4]. More outpatient care, fewer hospital beds and shorter inpatient stays redirect more rehabilitation and follow-up to primary care at an increasingly earlier stage of treatment [2,5]. Studies show that there is a considerable risk of adverse events in relation to the transition of patients between hospitals and primary care services and that information provided is often insufficient [6-8]. Thus, there is a growing need for better care coordination between primary and specialist health care services to ensure patient safety and continuity of care [1,9].

Many countries have focused attention on improving the coordination of their health and social care services [10,11]. In some European countries, models for hospital-at-home regimens have been developed as a beneficial alternative to inpatient care for selected patients [12,13]. Treatment and follow-up takes place in the patient’s home, with an ambulatory team from the local general hospital remaining responsible for patient care. Other models describe care pathways that aim to ensure adequate follow-up after discharge, involving both specialist and primary care services [14,15]. Most studies in the field evaluate models that have been initiated by specialist care services and are based on treatment of single diseases like stroke, heart failure and COPD [16-18]. Some studies describe care pathways for hospitalized elders more generally [19]. In these studies, hospital-based practice nurses or multidisciplinary teams are usually involved in the discharge process and for a limited post-discharge period. In Denmark an intervention was developed within primary care by GPs and home care services that reduced the risk of readmissions and improved medication control for newly discharged elderly patients [20].

Models have also been developed to improve the follow-up care of patients with chronic conditions in primary care. The Chronic Care Model has been introduced at several sites but targets mostly single diseases [21,22]. More recently, the Patient-Centered Medical Home model has been launched in the US [23].

Cultural differences between specialist care and primary care are not unknown [24]. However, we have not found studies investigating the potential implications that the different professional cultures might have on the process of developing care pathways across care levels.

In Central Norway a primary-care initiated project was set up where the main objective was better care coordination and follow-up during and following discharge from hospital to home by developing integrated care pathways. Being a cluster-randomised complex intervention, a process evaluation nested inside the trial was started in order to clarify causal mechanisms and to identify obstacles or other contextual factors contributing to the variation, success, or failure of the interventions [25]. The aim of this paper was to explore the process of developing the integrated care pathways that was going to be implemented in the project.

## Methods

This study used a qualitative design that included observations and interviews. The study was conducted from spring 2009 until spring 2010. It was approved by the Regional Committee for Medical and Health Research Ethics in Central Norway and the Ombudsman for Research at the Norwegian Social Science Data Service. The randomized trial was registered in Clinical Trials.gov NCT01107119.

All informants were informed about the study both in writing and orally by the first author and signed a written consent. They were informed that the interviews would be handled confidentially, that citations would be anonymous, and that they could ask for statements to be deleted.

### Setting

In Norway the general and university hospitals are owned by the government and managed through four regional health authorities. Primary care services, comprising for example general practitioners (GPs), home care services, nursing homes and community hospitals, are the responsibility of local authorities [26-28]. All citizens are entitled to have a GP who is responsible for providing general health care, including medical follow-up after discharge from hospital. These are usually organized as small private enterprises. Home care services are organized in district units employing nurses and aides who offer nursing and therapeutic procedures, medical services, personal care, social care and terminal care. Home care services may be offered several times a day and at night, when needed, and can even be provided continuously for 24 hours a day for shorter periods.

The framework for the project being studied was outlined by healthcare managers from the city of Trondheim in cooperation with St. Olavs Hospital and researchers from the Norwegian University of Science and Technology (NTNU) based on a literature search on care pathways across care levels for older patients.

Two general hospitals, one university hospital and six municipalities took part in the project, represented by people with experience in cooperation across care levels. Participants from all of the organizations met three times as part of a regional working group during a period of four months. They were given an introduction to the aims and tasks of the project and taught how to run the development process in their own organisations as local process facilitators (Table 1). They were guided by two supervisors from the Central Norway Regional Health Authority who had extended experience in coaching for developing clinical pathways within hospitals. The methods taught by the supervisors were based on the concepts Patient Process Redesign [29] and LEAN [30]. The participants in the regional working groups also formed three local working groups that met in between the regional sessions. These groups were led by one of the process facilitators and were organized around each of the participating hospitals and its adjacent municipalities. The local working groups were extended to involve additional nurses, physicians, physiotherapists, occupational therapists and participants from patient organizations. In addition, the local process facilitators arranged local meetings involving the staff at their workplace. The working groups were first asked to identify the risks for adverse events and potential obstacles during admission, discharge and follow-up at home, and to evaluate information flow, roles and responsibilities. Based on these analyses, they were challenged to develop care pathways for patients with COPD, heart failure and stroke. At the outset, the plan was to use the hospital-developed pathways and extend them into primary care by developing procedures for transition between the care levels and for follow-up in primary care.

**Table 1:** Local process facilitators (N = 27)

**Participants**	**Clinicians**	**Case handler**	**Managers**
Hospital nurses	10		
District nurses in home care services	8		3
Health and social administration, primary care		4	
Occupational therapists, primary care	2		

### Informants

Nineteen people (Table 2) were organized in three focus groups based on the local working groups. The informants were recruited by the first author and represented two patient organizations, five of the six municipalities, the three hospitals and the Central Norway Regional Health Authority. One small municipality was not represented in the interviews due to problems with capacity, and they temporarily pulled out of the project. The selection criteria were that the participants had participated actively throughout the development project in the regional and local working groups and that, in addition, all occupational groups were represented. Half of the informants had been local process facilitators, and two of them had managed the local working groups. All hospitals and municipalities were represented by at least two participants, and they made up about half of those who had been active in the regional and local working groups. Few GPs took part in the development process. However, collaboration between home care services and GPs was an important topic both in the process and the interviews. Therefore, a fourth focus group of four GPs was recruited; of these four, only one had taken part in the actual development process.

**Table 2:** Participants in the interviews (N = 23)

	**Participants**	**Age (mean/range)**	**Years of working experience**
Primary care	10	45 (30–62)	18 (6–37)
Hospital/Regional health administration	7	50 (36–59)	21 (9–36)
Patient organizations	2	67 (64–69)	
GPs	4	55 (51–61)	29 (25–33)

### Data collection

A semi-structured interview guide was used in the interviews (Table 3). The main question asked was: How did you experience the process of developing an integrated care pathway for older patients? Four focus groups were considered sufficient, as the representational spread was satisfactory, and the last interviews did not bring up new themes. All interviews were carried out by the first author. An independent co-moderator was present at two of the focus group interviews. The first author also participated as an observer at one regional meeting and at most of the meetings in the local working groups. Written material from all of the working groups, such as minutes, notes from flip-overs and proposed pathways, was collected and studied as well.

**Table 3:** Semi-structured interview guide

**Main question**	**Subordinate topics**
How did you experience the process of developing an integrated care pathway for older patients?	• Understanding of care pathways
	• Important topics in development work
	• Challenges regarding care pathways for older people
	• Responsibilities and collaboration in a care pathway
	• Expectations and attitudes in the development process
	• Challenges in the development process
	• Appraisal of the final solution

### Analyses

The interviews were recorded and transcribed verbatim by the first author. In the analyses we applied Malterud’s systematic text condensation, which is inspired by Giorgi’s phenomenological approach [31,32]. The authors studied the interviews independently in order to get a general sense of all the material and to identify the main themes. They then met to discuss and refine the identified themes. The first author then identified units of meaning related to the main themes, and the coding of these was discussed in subsequent meetings with the other co-authors. The original themes were re-evaluated throughout this process.

Additionally, six researchers familiar with qualitative studies and who had not been part of the project read the transcripts of the first focus-group interview independently and identified central themes. There were no major differences between these and the central themes already identified. The main results of the analyses were finally presented to informants from all geographical sites to uncover any apparent misunderstandings. The final analysis was studied and approved by the authors. The citations used are chosen to illustrate and complement the description of the findings.

## Results

The results were categorized into five main themes: The overall experience with the process is described under the heading “process experiences.” The details of the experience are described under the following headings: a tug of war between professional goals; disjointed collaboration in primary care; primary care perspectives gain ground; and merging of perspectives.

### Process experiences

The first regional meeting was described by the informants as confusing. For teaching the process method, all examples were taken from developing clinical pathways in hospitals, and the representatives from the municipalities were not able to relate the examples to their daily work. The participants from the hospitals and primary care understood the task at hand differently and struggled to understand each other’s point of view. They were able to identify several risks of adverse events, especially related to insufficient information flow both between the care levels as well as within primary care. However, on trying to develop a model for transition and follow-up, differences in professional objectives and perspectives between specialist care and primary care became very obvious and proved to be challenging. This influenced the first local meetings as well, and the participants could not agree on which perspective should form the basis of the care pathways.

At one stage we were uncertain if and how we could continue the process. We were miles apart. We didn’t understand each other’s point of view. (Nurse primary care, local process facilitator, city)

The project management was asked to intervene to get the process back on track, and this conflict of perspectives was a main theme in the next regional meeting. Furthermore, a geriatric nurse who had a great deal of working experience in both hospital and primary care joined the discussions and helped to bridge the gap between the participants from the hospitals and municipalities. These initiatives brought the process forward. The rest of the process was seen as constructive, and the dialogue was perceived as mutually respectful.

Gradually we accepted that each group had a completely different approach to the problem; that we came from different areas of expertise. The geriatric nurse helped us to speak the same language. That made things much easier, and then it became really fun. (Nurse primary care, local process facilitator, city)

### A tug of war between professional goals

The participants then started discussing discharge routines and follow-up for COPD, heart failure and stroke as proposed in the initial assignment. However, the strong focus on these single diseases was met with scepticism from most of the nurses in primary care. Their main concern, especially in the transition phase, was to assess the patient’s functional abilities and social situation in order to prepare for the necessary level of assistance and support needed at home.

I felt as if we were expected to be preoccupied with diagnoses. However, we were more concerned with the patient’s functional ability. (Nurse primary care, local process facilitator, city)

This made some hospital nurses feel that the district nurses were uninterested in the patients’ diagnoses. In the working group discussions, hospital nurses argued that many exacerbations of chronic conditions leading to hospital admissions might have been prevented had primary care done a closer follow-up of the disease. They said they were worried about the possible outcome of a care pathway that did not closely adhere to specific guidelines for each disease.

I wonder if a medical focus will be completely missing in the primary care program; it seems to have been given a back seat; it would appear that what I think is most important for the patient, follow-up of the disease, is wasted. (Hospital nurse, local process facilitator)

The representatives from the patient organizations acknowledged the perspectives from both parties telling that their attention changed from focus on disease in hospital into resuming daily activities when coming home.

These different perspectives caused confusion and consternation. However, even if the district nurses considered functional ability as the most important factor in the transition phase, they were also concerned about their patients’ chronic conditions in the follow-up at home. But they found that being restricted to assessment of single diseases for the three chosen diseases was unsatisfactory. Their patients rarely had only one single disease. In addition, diseases that were common in hospital might be infrequent for each nurse in primary care. They had to deal with the whole spectrum of diseases.

These concerns from the district nurses led to the proposal of developing discharge and follow-up routines applicable to most medical conditions, and common to all clinical hospital departments. This was met with astonishment by the majority of the hospital nurses.

There was an enormous difference between specialist care and primary care in how they approach care pathways. We found it difficult to understand why you [primary care] weren’t really interested in care pathways for specific diseases, and how you could think that one common care pathway might suit many diseases. (Supervisor, Regional Health Authority)

### Disjointed collaboration in primary care

The district nurses, in cities and rural areas alike, expressed a need for closer collaboration with both specialist care and GPs, as well as routines to regulate this collaboration. In recent years they had experienced that the medical needs of their care recipients had become increasingly more complex. They often felt that they had insufficient information about their patients to provide the necessary follow-up. They could therefore feel unsure as to what to observe and how to react to changes in the patients’ health.

When a patient is discharged the information we get is inadequate. And we can’t call the GP all the time either. To be able to know that we are doing a good job, nurses need to have a proper idea of the patient’s condition. I’m uncomfortable not having that type of control. (Nurse, primary care, rural area)

At the same time, the GPs complained that home care services reacted too slowly when patients’ health situations deteriorated.

And I’ve noticed that the district nurses aren’t always very good at monitoring patients. I have on several occasions experienced that they have seen the patient for one or two weeks without noticing that the patient is getting very ill. (GP, city)

The district nurses and GPs felt that there were organizational barriers to their collaboration such as geographic distance thus hindering a face-to-face relationship.

Nurses and doctors work closely and are on first-name terms when patients are in hospital. When the patients have had a minimal recovery, they are sent home. The possibilities for giving a good and coordinated follow-up then are completely different; in primary care, district nurses and GPs are geographically separated, might never have met each other and may not even know each other’s names. The present system means that all home care service units may have to collaborate with all GPs in the municipality. (GP, city)

### Primary care perspectives gain ground

During the development process, it became evident that the primary care perspective was gaining ground; this was also apparent from the interviews. Primary care representatives were in the majority because there were more municipalities than hospitals participating in the process. In addition, primary care was represented by three experienced managers of home care services, whereas the hospitals were not represented by the management level in any of the working groups. The challenges faced in daily work in primary care and home care services and in collaboration across care levels were therefore well illustrated in the discussions. Furthermore, the representatives from primary care appeared to have a more autonomous position. The hospital nurses did not feel that they had been given a mandate to propose changes in discharge routines for the whole hospital. In the discussions, they thus focused on the disease-related content of the care pathways. This was seen as being a very narrow approach by the district nurses.

The primary care representatives expected us to represent the whole hospital. We were shocked. We hadn’t been given a mandate to speak for the whole hospital. (Hospital nurse, local process facilitator)

The physicians played a lesser role in the process, both in the hospitals and in primary care.

Even when the doctors took part in the meetings, they were only there for some of the time, and they were focused on the follow-up of single diseases. (Nurse primary care, local process facilitator, rural area)

### Merging of perspectives

**Figure 1:**
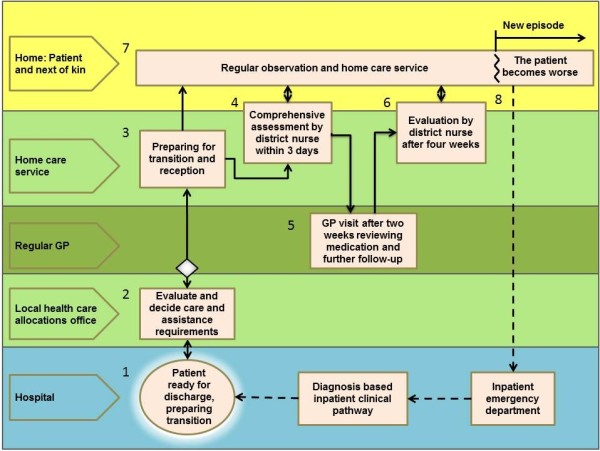
**Common care pathway for transition from hospital and follow-up of home care recipients. **The boxes represent procedures and checklists and the arrows the flow of information between involved parties. It starts with the patient being reported as ready for discharge and information is exchanged (**1 **and **2**). Home care services are established (**3**), and within three days a district nurse performs a thorough and structured assessment (**4**). The patient has a consultation with the GP 14 days after discharge (**5**), and a nurse or aide performs an extended assessment during the first four weeks (**6**). A daily care plan is continuously updated (**7**), and if the patient’s condition gets worse, the home care service has a routine for what to observe, whom to contact, and which information to pass on (**8**).

In the end the participants reached a consensus. The disease-based clinical pathways in the hospital were kept as before, while a common care pathway able to include most diagnoses was designed for the transition between hospital and primary care and for the follow-up in primary care (Figure 1). In the final phase of the process, the focus was on developing structures for collaboration and the flow of information. It became evident that there was a need for detailed descriptions of procedures, responsibilities and information flow with checklists for all situations that had been identified as critical in the risk-identification phase (Figure 1).

Quality control of a patient’s discharge and follow-up is simplified by using checklists no matter the diagnosis. They help us to remember to ask all the questions that need to be asked to ensure a proper follow-up. (Nurse primary care, local process facilitator, city)

## Discussion

Starting from the initial idea of using a disease-based model with several different care pathways, objections from primary care representatives led to the development of one common care pathway suitable for most common medical conditions covering admission and discharge from hospital as well as follow-up in primary care. However, different objectives and perspectives on patient care caused tension and obstacles between specialist care and primary care representatives in the joint process of designing clinical pathways. Table 4 summarizes how we interpret the differences that were uncovered. Primary care and hospital care pursue different professional goals and might not be fully aware of the needs and challenges of the other arena [24]. This study provides new insight into the ways in which different professional cultures play out in development processes.

**Table 4:** Cultural differences found between specialist care and primary care for patients with home care needs

**Activity**	**Specialist care**	**Primary care**
**Planning**	Short perspective – major changes in a short time	Long perspective – small changes over time
**Assessment**	Diagnosis with advanced technology	Functional ability, patient preferences and degree of self-management
**Diseases**	Attention to one disease at a time	Simultaneous attention to all of the diseases patients have; a majority of patients have multiple diseases
**Clinical guidelines**	Strong adherence to clinical guidelines	Clinical guidelines for multi-morbidity hardly exist
**Patient role**	Passive; health personnel decide what has to be done	At home the patient decides; focus is on resuming daily activities
**Decision making**	Often in teams, many involved, and in a confirmed hierarchical structure	Often by health personnel alone or by few; more autonomous

### Fragmentation in primary care

The increasing development of new specialties has contributed to fragmentation in health care [4,33]. Several publications have therefore pointed to primary care to ensure the continuity and integration of patients’ needs and care [34]. However, the interviews in this study confirmed that there is also significant fragmentation in primary care [28]. This problem has been accentuated as the home care services in Norway have developed from being primarily a social service providing practical help and support to becoming a healthcare service with an important role as well in advanced medical follow-up of chronic somatic and mental conditions [35,36]. However, better care coordination between GPs and home care services has been difficult to achieve thus far [37,38]. One important measure proposed in this study, therefore, was a mandatory GP visit for all patients who are discharged from hospital and need home care services (Figure 1).

### Clinical disease-based care pathways: sustainable in primary care?

The district nurses in our study were doubtful as to the usefulness of disease-based care pathways in primary care, as in their experience a large proportion of their patients had considerable co-morbidity. The prevalence of patients with multiple medical conditions increases with age and is substantial in the older population [39,40]. The specialist care informants gave an impression of district nurses not being interested in the treatment of the individual diseases. However, based on statements from the district nurses, there are reasons to believe that this was a misinterpretation. The impression was probably caused by the broad scope of measures that district nurses were concerned with in addition to treatment. They actually promoted a patient-centred approach that included functional ability, patient preferences, self-management and social needs [41]. They described that patients with chronic diseases have more common rather than differentiated needs. This, combined with the great prevalence of multi-morbidity, prepared the ground for one common clinical pathway for transition from hospital to follow-up in primary care. In the literature, care pathways based on a single medical condition are also found to be unsuitable for this patient group. This is because disease-based care pathways are founded in studies that largely exclude patients with co-morbid conditions [42]. Following clinical guidelines for individual diseases for patients with co-morbidity might even lead to potential treatment conflicts [43].

### The development process

Abandoning the disease-based model in favour of a patient-centred model was not an obvious result of the process. The supervisors from the regional health authority coaching the process were familiar only with diagnosis-based clinical pathways within hospitals, and the initial idea in the project was to develop care pathways for three diagnoses, which indicated that the representatives from the hospitals would be the experts. In addition, the GPs felt most comfortable with the disease-based model. However, the lack of participation by physicians in the working groups lessened their influence on the process.

Several other factors influenced the result. This was both a top-down and bottom-up process considered to be important in such development work [44], and the project had a broad representation from hospitals, primary care and patient organizations to ensure that all the different perspectives were taken into consideration. This is believed to be important both to overcome any asymmetry between primary care and the usually dominant hospital care [24] and to obtain a result with a patient perspective that could be sustainable both within specialist and primary care.

### Strengths and limitations

The results of this study came from experiences within a single regional setting. Any generalization of the findings should be made with caution. It is well known that there are major organizational differences in health care across countries that will influence and set limitations for what may be achievable and even legal. Norway has, compared to many countries, a well-developed primary care sector with an expenditure of approximately the same size as specialist care. However, the findings point to general challenges of cooperation in health care that have been thoroughly discussed in the literature [3,24,45].

A strength of the study is the use of triangulation: source triangulation by combining observations, written information from the workshops and interviews, and investigator triangulation by having several researchers with different backgrounds analyse the data and thus counteracting bias. The findings were finally validated by presenting the analyses to three of the informants, representing each of the three local working groups.

## Conclusion

In this study, it was found that the merging of primary care and specialist care perspectives led to a change from developing several separate, disease-based care pathways to one patient-centred care pathway suitable for most common diagnoses. The findings in this study challenge the sustainability of the current situation where most of the care pathways across specialist and primary care are disease based. The effect on patient outcome of a patient-centred care pathway for older patients needs to be studied.

## Abbreviations

COPD: Chronic obstructive pulmonary disease; GP: General practitioner; PC: Primary care.

## Competing interests

The authors declare that they have no competing interests.

## Authors’ contributions

TR, HG, and AG designed the research. TR collected data and drafted the manuscript. TR, AG, HG, and AS participated in the data analyses. All authors provided input on the manuscript and read and approved the final version.

## Authors’ information

TR: MD. Senior medical officer in the City of Trondheim

HG: MD, PhD. City Executive for Health and Welfare Services in Trondheim and Adjunct Associate Professor at Department of Public Health and General Practice, NTNU

AS: Sociologist, PhD. Professor at Department of Public Health and General Practice, NTNU

OS: MD, PhD. Professor at Department of Neuroscience, NTNU and head of Department of Geriatrics

AGM: MD, PhD. Professor at Department of Public Health and General Practice, NTNU and consultant at Norwegian Health Net.

## Pre-publication history

The pre-publication history for this paper can be accessed here:

http://www.biomedcentral.com/1472-6963/13/121/prepub
